# GITR signaling potentiates airway hyperresponsiveness by enhancing Th2 cell activity in a mouse model of asthma

**DOI:** 10.1186/1465-9921-10-93

**Published:** 2009-10-07

**Authors:** Alexandre C Motta, Joost LM Vissers, Renée Gras, Betty CAM Van Esch, Antoon JM Van Oosterhout, Martijn C Nawijn

**Affiliations:** 1 Laboratory of Allergology and Pulmonary diseases, Department of Pathology and Medical Biology, University Medical Centre Groningen (UMCG), Groningen University, Groningen, The Netherlands; 2 Pharmacology and Pathophysiology, UIPS, Faculty of Sciences, Utrecht University, Utrecht, The Netherlands

## Abstract

**Background:**

Allergic asthma is characterized by airway hyperresponsiveness (AHR) and allergic inflammation of the airways, driven by allergen-specific Th2 cells. The asthma phenotypes and especially AHR are sensitive to the presence and activity of regulatory T (Treg) cells in the lung. Glucocorticoid-induced tumor necrosis factor receptor (GITR) is known to have a co-stimulatory function on effector CD4^+ ^T cells, rendering these cells insensitive to Treg suppression. However, the effects of GITR signaling on polarized Th1 and Th2 cell effector functions are not well-established. We sought to evaluate the effect of GITR signaling on fully differentiated Th1 and Th2 cells and to determine the effects of GITR activation at the time of allergen provocation on AHR and airway inflammation in a Th2-driven mouse model of asthma.

**Methods:**

CD4^+^CD25^- ^cells were polarized *in vitro *into Th1 and Th2 effector cells, and re-stimulated in the presence of GITR agonistic antibodies to assess the effect on IFNγ and IL-4 production. To evaluate the effects of GITR stimulation on AHR and allergic inflammation in a mouse asthma model, BALB/c mice were sensitized to OVA followed by airway challenges in the presence or absence of GITR agonist antibodies.

**Results:**

GITR engagement potentiated cytokine release from CD3/CD28-stimulated Th2 but not Th1 cells *in vitro*. In the mouse asthma model, GITR triggering at the time of challenge induced enhanced airway hyperresponsiveness, serum IgE and *ex vivo *Th2 cytokine release, but did not increase BAL eosinophilia.

**Conclusion:**

GITR exerts a differential effect on cytokine release of fully differentiated Th1 and Th2 cells *in vitro*, potentiating Th2 but not Th1 cytokine production. This effect on Th2 effector functions was also observed *in vivo *in our mouse model of asthma, resulting in enhanced AHR, serum IgE responses and Th2 cytokine production. This is the first report showing the effects of GITR activation on cytokine production by polarized primary Th1 and Th2 populations and the relevance of this pathway for AHR in mouse models for asthma. Our data provides crucial information on the mode of action of the GITR signaling, a pathway which is currently being considered for therapeutic intervention.

## Background

Allergic asthma is an inflammatory disease characterized by reversible airway obstruction, and is associated with airways hyperresponsiveness (AHR) to bronchospasmogenic compounds, elevated allergen-specific IgE serum levels and chronic airway eosinophilia [1]. Th2 cells are known to be critical for the induction of allergic asthma manifestations by the production of IL-4, IL-5 and IL-13. Regulatory T (Treg) cells can counteract Th2 cell activity, and have the ability to suppress AHR and allergic inflammation upon allergen provocation in mouse models of allergic asthma. For instance, adoptive transfer of Treg cells into allergen-sensitized mice down-regulates asthma manifestations [2], while depletion of these cells exacerbates experimental asthma [3,4]. Interestingly, AHR was shown to be more sensitive than allergic airway inflammation to the number of regulatory T cells present in the lungs [5]. These data identify Treg cells as a potentially relevant target for therapeutic intervention in allergic asthma, in particular in case of persistent AHR, and Treg cell-based therapies are currently being considered for the treatment of this complex disease [6].

Glucocorticoid-induced TNF receptor family related protein (GITR) is a type I transmembrane protein and a member of the TNFR superfamily [7]. GITR is constitutively expressed to high levels on the cell surface of natural T regulatory (nTreg) cells [8,9]. In contrast, resting naïve CD4^+ ^T cells express very low levels of GITR, and its expression is strongly up-regulated following activation [9-14]. GITR stimulation was initially reported to abolish the suppressive properties of nTreg cells both *in vitro *and *in vivo *[9,15] However, this was later shown to be a T responder cell-intrinsic effect through the acquisition of resistance to Treg cell-mediated suppression [13]. In fact, GITR stimulation delivers a strong co-stimulatory signal to effector T cells, and increases proliferation and production of IL-2 of freshly purified mouse CD4^+^CD25^- ^cells stimulated *ex vivo *via CD3 and on mice splenocytes stimulated by CD3/CD28 or cognate peptides [10-12]. On the Treg cells, GITR stimulation also delivers a strong co-stimulatory signal, allowing IL-2 dependent proliferation of Tregs in the absence of TCR stimulation [16]. However, when GITR agonistic antibodies are added to mixed populations of CD4^+ ^T responder cells and CD4^+^CD25^+^FoxP3^+ ^Treg cells, the acquisition of resistance to suppression by the responder cells is the dominant effect, thereby functionally preventing the Treg suppressive effects [13,16].

While the effects of GITR stimulation on the total CD4^+ ^fraction are well characterized, studies aimed at dissecting the effects of GITR on polarized Th1 and Th2 effector cells yielded conflicting results [17,18]. On mouse primary CD4^+^CD25^- ^cells, addition of a GITR agonist antibody during *in vitro *differentiation into the Th1 or Th2 phenotype resulted in enhanced cytokine release from both Th1 and Th2 cells [17]. However, in fully polarized Th1 and Th2 cell clones, GITR triggering only enhanced Th1 cell proliferation at low cognate peptide concentrations, whereas for Th2 cells, GITR triggering retains its co-stimulatory effect on cell proliferation, irrespective of the dosage of the cognate peptide [18]. The effects on Th cell effector function or cytokine production was not analyzed in this study. To further investigate this issue, we evaluated the effects of GITR stimulation on primary and fully differentiated Th1 and Th2 cell populations, and show increased cytokine release from Th2 but not Th1 cells. Furthermore, to test the relevance of this observation *in vivo *we used an OVA-induced Th2-driven mouse model of asthma, characterized by AHR, induction of specific IgE and airway eosinophilia. We show for the first time that AHR is dramatically increased by GITR triggering at the time of allergen challenge, resulting in a left-shift of the response curve. In line with our *in vitro *data, this effect was associated with enhanced Th2 effector functions, such as increased secretion of IL-5, IL-10 and IL-13, and increased OVA-specific IgE levels in serum. Therefore, we conclude that GITR signaling during an ongoing immune response potentiates Th2 effector functions *in vivo*, resulting in an enhanced AHR and specific IgE levels in our mouse model of allergic asthma.

## Methods

### Animals

Animal care and use were approved by the Institutional Animal Care and Use Committee of the University of Groningen (IACUC-RuG). Specific pathogen-free (according to the Federation of European Laboratory Animal Science Associations) male BALB/c mice (6-8 wk old) were purchased from Charles River (Maastricht, The Netherlands) and housed in macrolon cages in a laminar flow cabinet and provided with food and water *ad libitum*. All experiments were performed using 6 mice per group.

### T lymphocytes skewing and stimulation in vitro

Unless specified, all recombinant cytokines and antibodies were purchased from Pharmingen BD. For Th cells *in vitro *differentiation, CD4^+^CD25^- ^cells were isolated from the spleen of naïve BALB/c by FACS sorting. CD4^+^CD25^- ^cells were then cultured in 96 wells plate (2 × 10^5 ^cells/well) at 37°C and 5% CO_2_, for 2 rounds of 4 days in RPMI medium containing 10% FCS and anti-mouse CD28 (1 μg/ml) on plate-bound anti-mouse CD3ε (2.5 μg in 50 μl PBS; 16 hours at 4°C). For Th1 polarization, recombinant mouse IL-12 (30 ng/ml), recombinant human IL-2 (10 U/ml) and anti-mouse-IL-4 (5 μg/ml) were added to the medium. For Th2 polarization, recombinant mouse IL-4 (40 ng/ml), recombinant human IL-2 (10 U/ml) and anti-mouse IFNγ (2.5 μg/ml) were added. Th1 and Th2 cells were then washed and cultured in 96 wells plates (2 × 10^5 ^cells/well) in RPMI 10% containing anti-mouse CD3ε (1 μg/ml), anti-mouse CD28 (1 μg/ml) and 10 μg/ml agonistic anti-GITR antibody (DTA-1, kindly provided by Dr. S. Sakaguchi) or control antibody (rat IgG). After 5 days, supernatant was collected and cytokines (IL-4 and IFNγ) levels were determined by ELISA.

### Mouse model of allergic asthma

Mice were sensitized intraperitoneally (i.p.) on days 0 and 7 with 10 μg OVA (grade V, Sigma-Aldrich, Zwijndrecht, Netherlands) in 0.1 ml alum (Pierce, Rockford, Illinois). After two weeks, sensitized mice were exposed to three OVA (10 mg/ml in saline) inhalation challenges for 20 min every third day. Mice were treated by i.p. injection of 1 mg DTA-1 or control antibody (rat IgG) 1 h before the first OVA inhalation challenge.

### Measurement of airway responsiveness in vivo

Several days before the first and twenty-four hours after the last OVA challenge, airway responsiveness was measured in conscious, unrestrained mice using barometric whole-body plethysmography by recording respiratory pressure curves (Buxco research systems, obtained through EMKA Technologies, Paris, France) in response to inhaled methacholine (Sigma-Aldrich). Airway responsiveness was expressed in enhanced pause (Penh), as described in detail previously [9]. The effective dose of methacholine that induced a half-maximal response, the ED_50 _value, was calculated after correction for baseline Penh values.

### OVA-specific IgE ELISA

After measurement of airway responsiveness *in vivo*, mice were sacrificed by i.p. injection of 1 ml 10% urethane in saline and were bled by cardiac puncture. Subsequently, serum was collected and stored at -80°C until analysis. Serum levels of OVA-specific IgE were measured by sandwich ELISA as described previously [10].

### Differential cell counts in the bronchoalveolar lavage fluid

Bronchoalveolar lavage (BAL) was performed immediately after bleeding of the mice by five injections of 1 ml saline (37°C) through a tracheal cannula into the lung. Cells in the BAL were centrifuged and resuspended in cold PBS. The total number of cells in the BAL was determined using a Bürker-Türk counting-chamber (Karl Hecht Assistent KG, Sondheim/Röhm, Germany). For differential BAL cell counts, cytospin preparations were made (15 × g, 5 min, 4°C, Kendro Heraues Instruments, Asheville, North Carolina). Next, cells were fixed and stained with Diff-Quick (Dade A.G., Düdingen, Switzerland). Per cytospin, 200 cells were counted and differentiated into mononuclear cells, eosinophils, and neutrophils by standard morphology and staining characteristics.

### Ex vivo lung cells re-stimulation

For lung cell re-stimulation, lungs were collected in PBS after sacrifice and single cell suspension were prepared. Lungs were minced using a scalpel and incubated for 1 h at 37°C in culture medium (RPMI 1640, 5% FCS, 1% glutamax I, gentamicin, all from Life Technologies, Gaithersburg, Maryland) containing DNAseI (0.5 mg/ml, Roche Diagnostics, The Netherlands) and Collagenase A (6.5 mg/ml, Roche Diagnostics). Lungs were then forced through a 70 μm mesh cell strainer, red blood cells were removed by lysis, and single-cell suspensions were washed twice in RPMI 5%. Lung cells were suspended in RPMI 10% containing 50 μM β-mercaptoethanol (Sigma-Aldrich) at a concentration of 6 × 10^5 ^cells/well in round-bottom 96-well plates (Greiner Bio-One GmbH, Kremsmuenster, Austria) in the absence or presence of 10 μg/ml OVA or plate-bound (2.5 μg in 50 μl; 16 hours at 4°C) rat anti-mouse CD3ε mAb. Each stimulation was performed in triplicate. After 5 days of culture at 37°C, the supernatants were harvested, pooled per stimulation, and stored at -20°C until cytokine levels were determined by ELISA.

### Cytokine ELISAs

IL-4, IFNγ, IL-5, IL-10 and IL-13 ELISAs were performed according to the manufacturer's instructions (all BD Pharmingen). The detection limits of the ELISAs were 60 pg/ml for IL-4, 32 pg/ml for IL-5, 15 pg/ml for IL-10 and IL-13 and 10 pg/ml for IFNγ.

### Statistical analysis

All data are expressed as mean ± standard error of mean (s.e.m.). After log transformation, airway responsiveness to methacholine was statistically analyzed by a general linear model of repeated measurements (ANOVA) followed by a post hoc comparison between groups using the Bonferroni method. Statistical analysis on BAL cell counts and lung tissue eosinophils were performed using the non-parametric Mann-Whitney U test (2-tailed). For ELISA, results were statistical analyzed using a Student's t-test (2-tailed, homosedastic). Results were considered statistically significant at the p < 0.05 level.

## Results

### GITR stimulation co-stimulates Th2 cytokine production

**Figure 1:**
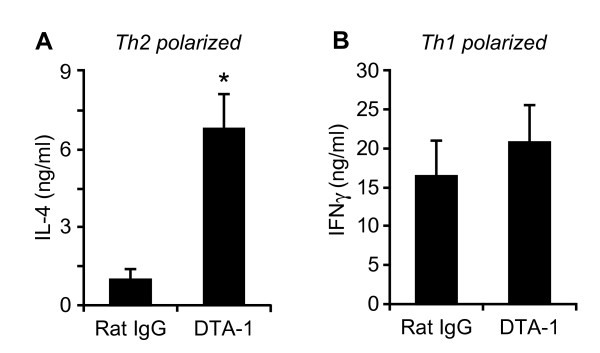
**Stimulation of GITR enhances Th2 but not Th1 cytokine production**. Co-stimulatory effect of DTA-1 on cytokine release upon CD3/CD28 activation of Th1 and Th2 lymphocytes. Th1- and Th2-polarized CD4^+ ^T cells were stimulated by anti-CD3ε (1 μg/ml) and anti-CD28 (1 μg/ml) in presence of 10 μg/ml DTA-1 or control antibody (Rat IgG). After 4 days of culture, supernatants were harvested and cytokines levels (**A**: IL-4 in Th2 polarized cells and **B**: IFNγ in Th1 polarized cells) were measured by ELISA. Results are expressed as the mean of 3 independent experiments ± SEM. *: p < 0.05 as compared to cells cultured in the presence of control antibody.

The effect of GITR signaling on Th1/Th2 cells has only been studied in fully polarized Th cell clones [18] and during Th1/2 cell differentiation [17], and this has yielded conflicting data regarding the effect of GITR signaling on Th1 cells. To further investigate the effects of GITR signaling on fully differentiated primary Th1 and Th2 cell populations, we isolated CD4^+^CD25^- ^cells, polarized these in two rounds of stimulation and re-stimulated the cells in the presence of DTA-1 (GITR agonist antibody). We find that GITR stimulation induced increased IL-4 production from Th2 cells (Figure 1A) but did not further enhance IFNγ production from Th1 cells (Figure 1B). These data indicate that primary, fully differentiated Th2 but not Th1 effector cell populations are sensitive to GITR-dependent co-stimulation of cytokine production.

### DTA-1 treatment enhances airway hyperresponsiveness in a mouse model of asthma

**Figure 2:**
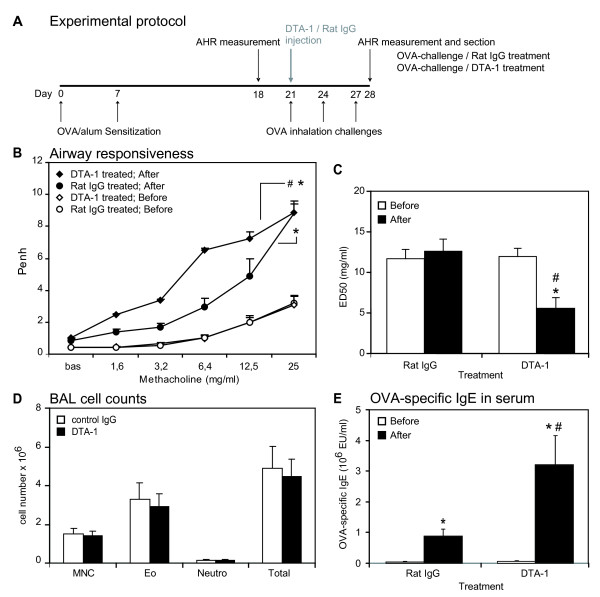
**GITR stimulation aggravates AHR and serum IgE responses in a mouse model of asthma**. A. OVA-induced asthma model. Sensitization: i.p. injection of OVA/Alum (day 1, 7). Challenge: OVA inhalation (day 21, 24, 27). DTA-1 treatment: 1 hour before the first OVA challenge (day 21). AHR was measured before (day 18) and after (day 28) OVA challenges. BAL, blood and lungs were collected (day 28). One experiment is shown out of two independent experiments performed (giving similar results) with 6 mice per group in each experiment. B. Airway responsiveness to methacholine measured in OVA-sensitized mice before (O: control antibody; ◊: DTA-1) and after (black circle: control antibody; black diamond: DTA-1) OVA challenges, expressed as enhanced pause (Penh). Bas: baseline Penh. *: *P *< 0.05 as compared to before OVA challenges and #: *P *< 0.05 as compared to control antibody treatment. C. ED_50 _values of the methacholine dose-response curves before (white bars) and after (black bars) OVA challenges. *: *P *< 0.05 as compared to before OVA challenges and #: *P *< 0.05 as compared to control antibody treatment. D. Numbers of leukocytes in the BAL after OVA inhalation in mice treated with control antibody (white bars) or DTA-1 (black bars). MNC: mononuclear cells; Eo: eosinophils; Neutro: neutrophils; Total: total cell counts. E. Serum levels of OVA-specific IgE in serum, before (white bars) and after (black bars) OVA challenges in DTA-1 or control antibody-treated mice. Results are expressed in experimental units (EU/ml). *: *P *< 0.05 as compared to before OVA challenges and #: *P *< 0.05 as compared to control antibody treatment.

To test whether our *in vitro *observations are relevant to Th2 cell effector functions *in vivo*, we studied the effects of GITR stimulation in a Th2-driven mouse model of asthma (Figure 2A). In this model, OVA airway challenges in sensitized mice triggers AHR, airway eosinophilia and an OVA-specific IgE response in a Th2-dependent way. To determine the effect of GITR activation on AHR, sensitized mice were treated with 1 mg DTA-1 or IgG control antibody 1 h prior the first OVA challenge. AHR to increasing doses of methacholine was measured prior to the first OVA challenge and 24 h after the last of a series of three OVA challenges (Figure 2A). Compared to the responses before allergen challenge, all OVA-challenged mice showed marked AHR (Figure 2B). However, DTA-1 administration induced a further increase in the AHR to methacholine as evident from the left-shift of the Penh curve compared to control antibody-treated mice, resulting in a statistically significant decrease of the methacholine ED50 (Figure 2B, C).

### DTA-1 treatment does not affect lung eosinophilia

In the mouse asthma model, a strong eosinophilic airway inflammation is induced upon allergen challenge. Indeed, mice challenged with OVA showed a characteristic eosinophilia compared to controls, but DTA-1 treatment did not further increase lung eosinophilia compared to control antibody (Figure 2D). This result was confirmed by lung tissue histology (data not shown). Similarly, the amount of infiltrated lymphocytes was not modified by DTA-1 treatment.

### DTA-1 treatment increases levels of OVA-specific IgE

Another important characteristic of our asthma model is the induction of OVA-specific IgE responses. OVA challenge induced a statistically significant increase in specific IgE levels and DTA-1 treatment strongly potentiated IgE levels as compared to control antibody (Figure 2E). Taken together, these results suggest an increase of the Th2 response upon GITR stimulation.

### Lung T cells cytokines production

**Figure 3:**
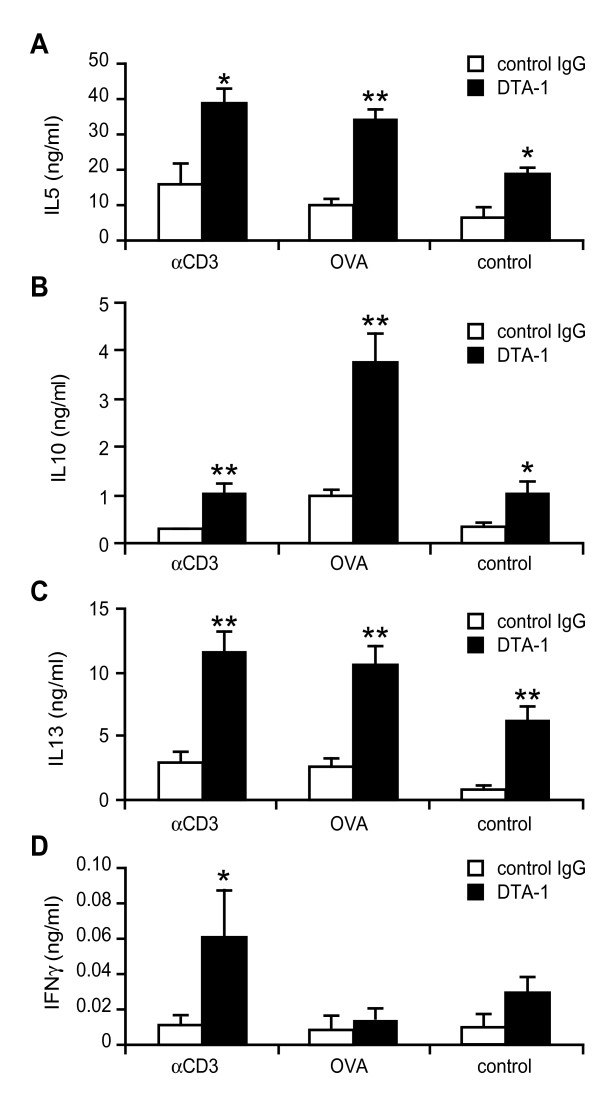
**GITR stimulation *in vivo *induces enhanced Th2 cytokine production *ex vivo***. Effect of treatment with DTA-1 *in vivo *on T-lymphocyte cytokine production *ex vivo*. Lung lymphocytes derived from OVA challenged mice treated with DTA-1 (black bars) or control antibody (white bars) were cultured for 5 days in medium only (control), or in presence of plate-bound anti-CD3ε or soluble OVA (10 μg/ml). (A) IL-5 production, (B) IL-10 production, (C) IL-13 production and (D) IFNγ production in ng/ml. *: *P *< 0.05 and **:*P *< 0.01 as compared to control antibody treatment. The results shown are from one experiment out of two independent experiments performed (giving similar results).

To verify the involvement of Th2 cells in the observed effects of DTA-1 on AHR and IgE levels, lung cells were isolated following sacrifice and cultured *ex vivo *in medium alone or re-stimulated by plate-bound CD3ε or soluble OVA. After 5 days of culture, the levels of Th2 cytokines IL-5, IL-10, IL-13 and IFNγ in supernatants were measured by ELISA. As shown in Figure 3, lung cells isolated from DTA-1-treated mice produced higher amounts of IL-5, IL-10 and IL-13 upon stimulation (either polyclonally 'CD3' or antigen-specifically 'OVA') as compared to cells isolated from control-antibody treated mice. Interestingly, these differences could also be found in cells that did not receive any further stimulation *ex vivo *('control'), indicating that the observed cytokine production was at least in part due to the *in vivo *activation of the isolated cells. The levels of the Th1 cytokine IFNγ were very low (Figure 3D). Upon antigen-specific ('OVA') re-stimulation *ex vivo*, IFNγ production was similar between DTA-1 and control treated mice (Figure 3D), in line with our *in vitro *observations (Figure 1). However, upon polyclonal *ex vivo *re-stimulation, IFNγ levels were slightly increased in cells isolated from DTA-1 treated mice, indicating that a non-antigen specific T cell population might have been affected by the treatment. Taken together, these data indicate that the DTA-1 treatment resulted in an exacerbated activity of the antigen-specific Th2 cells *in vivo*.

## Discussion

In this study, we show that GITR exerts a co-stimulatory effect on cytokine production of fully polarized Th2 but not Th1 cell populations *in vitro*. In agreement with these *in vitro *observations, GITR triggering at the time of allergen challenge in a mouse model of allergic asthma increased AHR and levels of OVA-specific IgE in serum.

The effects of GITR stimulation on T cell responses are dual. It is generally accepted that GITR engagement on naïve or effector T cells provides resistance to Treg cell-mediated suppression, as well as delivering a co-stimulatory signal leading to enhanced proliferation and cytokine production [16]. When associated with CD3 stimulation, naïve mouse CD4^+^CD25^- ^cells show higher proliferation upon GITR signaling by DTA-1 or GITR ligand [10,12]. At the same time, GITR signaling also delivers a strong co-stimulatory signal for Treg cell proliferation [16].

The direct effects of GITR signaling on Th1 and Th2 effector functions have not been characterized in great detail. One study showed an up-regulation of cytokine production from both Th1 and Th2 cell populations differentiated *in vitro *in the presence of GITR agonistic antibody [17]. In contrast, when fully polarized Th1 and Th2 cell-lines are stimulated through TCR by cognate peptide presentation, the co-stimulatory effect of GITR signaling on Th1 cell proliferation can only be seen at low peptide concentrations, while in Th2 cells GITR signaling has a co-stimulatory effect on cell proliferation irrespective of the strength of the TCR signal, suggesting that GITR exerts a differential effect on Th1 and Th2 cell proliferation [18]. In this latter study, however, effector functions of Th cell subsets were not analyzed. Here, we show for the first time that GITR signaling has no co-stimulatory effect on cytokine production by primary, fully polarized Th1 cell populations *in vitro*.

The effect of GITR signaling on T cell responses *in vivo *has been studied in considerable detail. For instance, it has been reported that the progression of Th1-driven acute graft versus host disease is inhibited by treatment with a GITR agonist antibody (DTA-1), and that this effect is dependent on the inhibition of Th1 cells [19]. In contrast, several other studies have reported aggravating effects of DTA-1 treatment on mouse disease models with a strong Th1 component, such as autoimmune gastritis [9], autoimmune encephalomyelitis (EAE) [11] or HSV infection [20,21]. Although the enhanced *in vivo *Th1 activity in these studies might be explained by indirect effects of the DTA-1 antibody treatment on Treg cells [16], Treg depletion did not alter the DTA-1 effect in the EAE model [22]. In contrast to Th1-driven disease models, the role of GITR triggering in Th2-driven disease models has not been studied extensively *in vivo*. In one study it was shown that DTA-1 administration during allergen challenges exacerbated eosinophilic airway inflammation and OVA-specific IgE responses, indicating that Th2 effector functions were augmented *in vivo *as well [17].

We show that GITR treatment at the time of allergen challenges increases AHR to methacholine in our mouse asthma model, as shown by the left-shift of the AHR response curve. Clinically, AHR is the most characteristic feature of allergic asthma and is the main factor of morbidity in asthma patients. This is the first time that GITR triggering is reported to have a direct effect on this critical parameter for lung function. The effect of GITR triggering on AHR likely reflects enhanced Th2 effector function leading to an increased IL-13 production from lung cells, which has been shown to be the main effector cytokine inducing AHR [23]. From our data, it is not possible to determine whether the increased Th2 cytokine production by lung cells was due to a higher number of Th2 cells recruited to the lungs or an enhanced cytokine production by individual Th2 cells. Nevertheless, the latter seems to be more likely when combining our *in vitro *cytokine measurement data with the fact that the amount of lymphocytes in the BAL did not differ between control and DTA-1-treated mice.

Surprisingly, eosinophil infiltration in the lung was not further increased by DTA-1 treatment although IL-5 production by lung cells was enhanced. This could be explained by the concomitant increase of IL-10 production by these cells, which has been shown to antagonize eosinophil recruitment but potentiate AHR in a similar mouse model of allergic asthma [24]. These observations are in line with several studies reporting dissociation between AHR and airway eosinophilia in mouse asthma models [25,26].

Our results on the effect of DTA-1 treatment on airway eosinophilia are in contrast to the study of Patel *et al*. mentioned above where lung eosinophilia was increased [17]. Several differences between the two experimental asthma protocols used in their and our studies can explain these differences. First, we used male mice, which display higher levels of AHR in mouse asthma protocols than do females, whereas the Patel study used female mice, which display stronger parameters of allergic inflammation (IgE, airway eosinophilia) than do male mice [27]. Second, the amount of OVA used for the sensitization and the administration route for the challenges were different. Finally, Patel *et al*. administered DTA-1 at 2 different time points, 1 day before the first challenge and 1 h before the second challenge [17], whereas we only gave DTA-1 once 1 h before the first challenge. In our model, we do observe a strongly increased OVA-specific IgE response in serum after DTA-1 treatment, indicating that the augmentation of Th2 effector function is consistent between the two studies.

It is possible that DTA-1 treatment had an effect on the Treg cell subset in our mouse model of asthma [4,6]. In our experiments, we cannot exclude that the GITR-mediated increase of asthma manifestations was partly due to an effect on Treg cell. In fact, the selective effects we observe on AHR but not on airway eosinophilia are in line with a decreased number or activity of Treg cells in the lungs [5]. Nevertheless, our *in vivo *data on serum IgE and Th2 cytokine production in the lung seem to indicate a potentiation of Th2 effector functions in accordance with our *in vitro *observations. In our experiments we cannot distinguish whether this augmented Th2 effector activity is the result of a direct effect of GITR signaling on Th2 cells, an indirect effect of GITR signaling on Tregs, or a combination of the two.

In conclusion, we show that activation of GITR during allergen exposure can aggravate AHR in a mouse model of allergic asthma, which seems to be associated with increased Th2 cell activity in the lungs and elevated serum IgE responses. Our data bear relevance to the understanding of the mode of action of the GITR in cell-mediated immunity, a pathway which is currently considered for potential therapeutic intervention [28].

## Conclusion

Activation of GITR during allergen provocation induces an exacerbated Th2 cell response in the lungs and aggravates airway hyperresponsiveness to methacholine in a mouse model of allergic asthma, as shown by a left shift of the AHR response curve to methacholine.

## Abbreviations

AHR: airways hyperresponsiveness; GITR: Glucocorticoid-induced TNF receptor family related protein; OVA: Ovalbumin; Penh: enhanced pause; Treg: Regulatory T cell.

## Competing interests

The authors declare that they have no competing interests.

## Authors' contributions

ACM performed the *in vitro *experiments, contributed to the *in vivo *experiments and drafted the initial version of the manuscript. JLMV contributed to the *in vitro *and *in vivo *experiments. RG and BCAMvE contributed to the *in vivo *experiments. AJMvO conceived of the study, participated in its design and coordination. MCN contributed to the *in vivo *experiments, participated in the design and coordination of the study and drafted the final manuscript. AJMvO and MCN share senior authorship. All authors have read and approved the final manuscript.
